# Redomesticating Almond to Meet Emerging Food Safety Needs

**DOI:** 10.3389/fpls.2020.00778

**Published:** 2020-06-12

**Authors:** Thomas M. Gradziel

**Affiliations:** Department of Plant Sciences, University of California, Davis, Davis, CA, United States

**Keywords:** allergen, Salmonella, aflatoxin, domestication-bottleneck, germplasm, introgression, immunoreactivity

## Abstract

Almond is a desirable and high-quality food source where the presence of nut allergens and a vulnerability to aflatoxin and Salmonella contamination represent threats to consumer safety. In 2019, over 1 billion kg. of almonds, representing over 80% of the world total, were produced in California from a relatively few varieties with a very narrow genetic base. To address emerging needs mandated by cultural and climate changes, new germplasm has been introduced combining peach as well as wild peach and wild almond species. Advanced breeding selections incorporating exotic germplasm into a genetic background compatible with commercial production in California have demonstrated sizable reductions in level of kernel immunoreactivity as well as opportunities for improved control of aflatoxin and Salmonella. Breeding strategies employed include direct selection for reduced kernel immunoreactivity from an introgression enriched germplasm, the integration and pyramiding of resistance to multiple components of the aflatoxin disease-insect complex, and introduction of novel nut and tree traits to facilitate mechanized catch-frame field harvesting to avoid contamination with soil-borne pathogens such as Salmonella and *Escherichia coli*, as well as agrochemical residues.

## Introduction

The almond [*Prunus dulcis* (Miller D.A. Webb) syn. *Amygdalus dulcis* Mill., *Prunus amygdalus* (L.) Batsch, and *Amygdalus communis* L]. represents a nutritious, desirable, and relatively non-perishable food item as well as a durable propagation source for expanding plantings. These qualities made it commercially as well as horticulturally desirable, even in ancient times. The wild almonds traded and consumed by early civilizations were represented by over 30 species of diverse quality, morphology, and geographic origin (Zeinalabedini et al., 2010). Almond’s widespread desirability and easy transportability appear to have made it an important commodity in prehistoric trade in Asia, North Africa, and Europe (Zohary et al., 2012), eventually leading to the establishment of an evolving commercial standard as well as a new species: the cultivated sweet almond (*Prunus dulcis*) probably selected by prehistoric societies from desirable interspecific hybridizations (Gradziel, 2017). Germplasm erosion resulted from domestication bottlenecks as well as subsequent regional planting practices. Almonds were originally planted as genetically diverse seedling orchards where the relatively common bitter seedlings would either be retained as a source of bitter almond extract for use in making marzipan, etc., or grafted to more desirable sweet varieties. In a major advancement for almond genomics, Sánchez-Pérez et al. (2019), after successfully sequencing the almond genome, have identified the genetic mutation controlling kernel sweetness that allowed almond’s domestication as a food crop. The higher value of established and so well-characterized sweet-kernel varieties led to their occupying a greater proportion of subsequent plantings, contributing to inbreeding and germplasm loss. In California, while over 100 geographically diverse varieties were grown in the early 1900s, the varieties “Nonpareil” and “Mission” dominated plantings by mid-century (Gradziel et al., 2017). Of the current plantings of approximately 540,000 hectares, “Nonpareil” remains the dominant variety with most of remaining pollenizer varieties (almond is self-sterile), having “Nonpareil” and “Mission” as direct parents (Gradziel and Martínez-Gómez, 2013).

In addition to domesticated almond, sweet kernels of apricots (*Prunus armeniaca* and *P. mandshurica*), plums (*Prunus domestica*), peaches (*Prunus persica*), and wild almond species would have been consumed in ancient times as they are to this day (Gradziel, 2011). The term “badam,” which, when used alone refers to almond in a wide range of Asian languages can also refer to the edible kernels of other Prunus. For example, “tao’ze” badam refers to peach kernel in western China and “khasta badam” to peach or apricot kernel (sometimes called “poor man’s almond”) in India. Because of the absence of well-defined quality evaluation guidelines for almond and related nuts, most attempts to define kernel ideotypes consider only size, shape and kernel bitterness (R Socias i Company et al., 2008).

Due to its high nutritional and eating quality, almonds have become a valued component of many diets. Almond is an important source of macro-nutrients such as lipids, proteins, fiber and minerals, and is increasingly being recognized as an important source of the phytonutrients vitamin E (α-tocopherol), folate, and oleic acid. As with many other nut crops, almond are also important sources of food allergens and potential contamination with aflatoxins and human pathogens such as *Salmonella* and *Escherichia coli*, as well as agrochemical residues that can pose serious health risks for consumers.

In this study, previously published and unpublished data on allergenicity and susceptibility to aflatoxin and Salmonella contamination are evaluated in breeding germplasm derived from interspecies crosses in order to determine whether re-synthesized or redomesticated germplasms can be identified with improved nutritional and food safety qualities.

## Materials and Methods

A diverse germplasm, including 10 commercial varieties, seven related *Prunus* species and 47 inter-species hybrids and introgression lines from the University of California, Davis (UCD) genetic improvement program that had been selected for self-fertility and local adaptability but not kernel nutrient quality were evaluated for kernel and nut quality, soluble protein, and kernel immunoreactivity (Table 1). Commercial varieties evaluated originated in California, Spain, France and Italy, and include the recently released “Sweetheart” variety that originated from an intraspecific hybridization between “Mission” almond and “Lukens Honey” peach followed by three successive backcrosses to almond (“Mission” almond × *P. persica)BC3*. Evaluated germplasm included 6 additional introgression-derived selections newly released for grower testing. Commercial varieties and the introgression-derived “Sweetheart” variety were also evaluated for resistance to contamination by aflatoxin and *Salmonella* spp. The main commercial variety “Nonpareil” was included in all evaluations as the industry standard.

**TABLE 1:** Nut and kernel characteristics, including ELISA immunoreactivity values, for an intra- and interspecific almond breeding germplasm.

No.	Genotype	Origin	Expected Percent Almond	Kernel Length (mm.)	Kernel Width (mm.)	Kernel Breadth (mm.)	Kernel Mass (*g*.)	Nut Length (mm.)	Nut Width (mm.)	Nut Breadth (mm.)	Nut Mass (*g*.)	Soluble protein (*g*./100*g*)	ELISA
	**Commercial varieties**												
9	Sonora	Almond variety (United States)	100	27.7	13.1	7.8	1.52	37	18.9	12.7	2.25	22.07	0.74
10	Nonpareil	Almond variety (United States)	100	24.7	13.5	7.9	1.31	34.3	17	15	2.2	23.07	1.02
19	Mission	Almond variety (United States)	100	20.8	12.4	8.9	1.04	27.9	19.8	15.8	2.55	19.17	0.86
20	Chips	Almond variety (United States)	100	21.5	12.7	8.2	0.96	28.7	19.5	14.7	2.02	26.46	1.68
21	Kahl	Almond variety (United States)	100	26	12.1	8	1.2	34.3	17	15	2.2	26.29	1.22
22	Ferragnes	Almond variety (France)	100	26.8	14.2	8.3	1.48	36.4	23.1	17	4.09	19.37	1.56
24	Winters	Almond variety (United States)	100	26.3	11.9	8.1	1.21	36.4	19.3	14.1	2.09	22.37	1.05
25	Marcona	Almond variety (Spain)	100	22	17.3	8.8	1.55	29.4	25.8	19.6	5.55	22.22	0.88
26	Tuono	Almond variety (Italy)	94	26.4	16.3	8.2	1.58	38.4	27.7	18.3	5.45	17.14	0.52
	**New releases**												
23	Sweetheart	Almond variety (Peach × Almond)BC3	94	19.1	12.5	8.8	0.98	22.5	19	14.3	1.54	25.52	1.73
29	UCD,8–160	Nonpareil × 97,1–232	91	28.6	14.2	8.6	1.77	38.5	22.5	15.4	2.96	19.84	1.20
30	UCD,8–201	Nonpareil 97,1–232	91	24.1	13	8.1	1.26	32.1	21.5	14	2.06	15.81	1.67
41	UCD,2–3	[Almond × (*P. webbii* × *P. persica*)] (BC3)	94	23.9	11.6	9	1.17	31.8	22.4	14.6	4.74	19.89	1.93
42	UCD,8–27	[Almond × (*P. webbii* × *P. persica*)] (BC3)	94	24.3	12.1	8.6	1.2	30.4	20.9	14.2	3.36	23.92	0.55
54	UCD,2–240	(Nonpareil × *P. webbii*) BC3	94	23.8	12.6	9.5	1.28	30.3	24.3	14.3	5.62	22.22	0.40
64	UCD,3–40	[Almond × (*P. webbii* × *P. persica*)] (BC2)	88	33.3	15.1	8.7	2.08	39.2	29.6	18.8	9.21	25.31	0.90
	**Related species**												
15	40A–17	Peach (*P. persica*; bitter seed)	0	13.4	7.2	3.4	0.11	24.3	16.8	12.5	1.81	23.74	0.51
16	Andross	Peach (*P. persica*; bitter seed)	0	17.8	11.4	3.9	0.36	35.3	26.1	19.5	6.21	20.65	0.39
27	P11–58	*P. mira* (bitter seed)	0	14.5	9.9	4.3	0.29	26.6	17.8	12.8	2.48	23.39	0.53
43	A7–28	*P. webbii* (bitter seed)	0	18.4	9.1	6.3	0.49	25.7	14.1	10.2	1.39	21.04	0.88
55	A7–23	*P. argentea* (bitter seed)	0	13.4	9.7	6	0.37	19	15.3	12.1	1.47	17.28	0.61
61	A10–4	*P. bucharica* (bitter seed)	0	14.3	6.6	4.7	0.21	19.1	10.3	7.4	0.58	20.94	0.59
62	A2–11	*P. tangutica* (bitter seed)	0	13.4	10.3	8.3	0.49	16.5	15.2	12.4	1.34	25.44	0.70
63	A7–25	*P. webbii* (bitter seed)	0	20.4	11.8	7.3	0.82	29	18.3	13.7	2.93	19.09	0.51
	**Interspecies hybrids**												
1	F5,4–10	*P. webbii* × (Nonpareil × *P. persica*)	25	19.7	11.9	7.2	0.78	27.5	18.3	12.8	2.69	22.12	0.53
6	F5,20–42	Padre × F5,4–10	62	21.4	12.1	8.2	1	26.8	17.9	14	1.87	16.72	0.65
7	F8N,6–68	F5,4–10 × Solano	62	21.6	12.5	7.2	0.96	30.7	19.9	14.4	1.89	23.47	0.88
8	F8N,7–4	F5,4–10 × Sonora	62	22.7	10.7	6.2	0.76	32	16.1	10.7	1.17	19.52	0.65
12	8010–22	Nonpareil × F5,4–10	62	24.6	12.5	7.1	1.05	37.6	19.3	14.1	1.9	21.06	2.09
17	SB13,25–75	Nonpareil × F5,4–10	62	23.1	12.5	7.8	1.17	30	22.3	14.7	2.56	22.18	1.78
32	A13–1	*P. persica* × *P. davidiana* (bitter seed)	0	13.8	11.4	6.1	0.46	21.5	20.7	17.8	3.83	23.41	0.45
33	Hansen2	Almond × *P. persica* Rootstock	50	28	15.7	7.3	1.44	44.1	28.5	18.3	9.07	12.35	1.57
34	Hansen5	Almond × *P. persica*	50	23.8	13.9	7.5	1.12	34.5	24.6	18.9	7.44	21.06	0.66
35	Nickels	Almond × *P. persica*	50	23.9	16.4	8.8	1.53	36.9	28.7	20.9	9.18	13.79	0.75
39	F10D,1–26	Nonpareil × F5,4–10	62	23.1	14.2	6.9	1.11	30.8	24.8	15.8	3.88	17.64	1.61
	**Interspecific introgressions**												
2	F5,6–13	(Mission × *P. fenzliana*) BC1 × Sonora	88	22.1	10.8	6.7	0.84	32	17.3	10.5	1.66	25.6	0.95
3	F5,6–1	(Mission × *P. fenzliana*) BC2	88	23	14.6	7.4	1.33	33.8	23.7	16.8	5.08	25.88	0.92
4	F5,13–54	(Mission × *P. fenzliana*) BC1 × Sonora	88	23.7	11.9	8.3	1.05	37.2	19.5	16.7	2.94	16.28	0.70
5	F5,10–9	(Mission × *P. fenzliana*) BC1 × Sonora	88	21.1	12.2	7	0.82	27.3	18.8	14.2	3.08	18.11	0.61
11	SB13,54–39E	(Nonpareil × *P. persica*) BC3	94	16.9	10.2	8.2	0.7	26.2	15.8	12.3	1.05	21.51	1.96
13	F10C,12–28	(Nonpareil × *P. persica*) F2	50	20.2	13	9	1.08	35.1	23.9	18	4.96	19.32	1.76
14	F10C,20–51	(Nonpareil × *P. persica*) F2 (bitter seed)	50	25.1	12.6	7.3	1.1	35.1	21.3	15	2.43	23.87	0.56
18	F5,16–60	(Mission almond × *P. argentea*) F2	50	23.8	11.1	7.3	0.87	32.9	17.1	11.9	1.56	24.08	0.44
28	97,1–232	SB13,25–75 × Winters	81	23.6	13.4	8.2	1.29	31.3	20.4	13.5	2.27	20.61	2.06
31	2004,9–1	Nonpareil × 97,1–232	91	25	13.5	7.5	1.24	34.3	23.8	18.1	3.15	14.54	1.89
36	2005,20–192	(Nonpareil × *P. persica*) BC3	94	20.6	14.6	7.4	0.99	37.1	26.5	19.3	7.31	23.91	0.63
37	F10D,3–7	[Almond × (*P. webbii* × *P. persica)*] (BC1)	75	20.5	10.6	6.7	0.69	26.3	16.6	12.6	1.41	15.35	0.42
38	F10D,2–18	Nonpareil almond× *P. webbii* (BC1)	75	19	10.8	8.5	0.8	24.9	17.5	13.1	1.95	22.4	0.76
40	F10D,3–23	Padre almond × *P. webbii* (BC1)	75	20.4	11.9	7.7	0.84	27.5	19.8	13.4	2.32	14.48	1.49
44	F5,4–42	Almond × *P. webbii* (F2)	50	18.5	9.5	6.7	0.55	26.8	15	10.8	1.96	25.8	0.64
45	F10D,3–15	Almond × *P. webbii* (F2BC1)	75	24	12.9	7.2	0.96	33.3	21	14.6	4.1	18.58	0.33
46	F10D,1–22	Almond × *P. webbii* (F2BC1)	75	21.6	12.7	7.7	0.97	28.9	21.4	15.2	2.45	21.05	1.78
47	F10D,1–4	Almond × *P. webbii* (BC1)	75	23.1	11.9	7.6	0.95	30.8	18.1	13.3	1.94	20.5	1.32
48	F10D,1–2	Almond × *P. webbii* (BC1)	75	20.8	12.2	7.2	0.84	30	19.8	14.2	1.59	20.4	0.68
49	F10D,3–2	Almond × *P. webbii* (BC1)	75	19.7	11.1	7	0.77	30.6	17.8	13.6	1.53	17.84	0.66
50	F10D,2–5	Almond × *P. webbii* (BC1)	75	20.8	9.8	8.1	0.76	28.7	14.6	11.3	1.23	17.99	0.47
51	F10D,3–26	Almond × *P. webbii* (BC1)	75	24.1	11.4	7.5	0.93	33.6	20.3	14.4	3.23	21.17	1.06
52	F10D,3–13	Almond × *P. webbii* (BC1)	75	19.4	12	8	0.83	25.4	19.1	13.7	1.85	17.07	0.47
53	F10D,3–24	Almond × *P. webbii* (BC1)	75	19.3	13.2	6.1	0.71	25.7	19.5	13.3	2.66	13.39	1.27
56	F10D,3–3	Almond × *P. argentea* (BC1)	75	23.4	12.4	7	0.96	29.6	18.6	13.8	1.88	17.47	0.26
57	F10D,2–12	Almond × *P. fenzliana* (F2)	50	20.6	10.8	7	0.77	26.5	16.1	11.5	1.41	21.38	1.53
58	F10D,2–14	Almond × *P. fenzliana* (F2)	50	22.3	11.4	8.4	1.03	30.6	16.5	11.3	4.54	19.21	1.66
59	F10D,2–3	(Mission × *P. fenzliana*) BC1 × Sonora	88	21.8	13.2	8.9	1.13	27.6	20.1	16.3	3.24	20.71	1.56
60	F10D,3–50	Almond × *P. fenzliana* (BC1)	75	27.3	13.9	8.8	1.59	36.2	19.3	13.3	2.37	15.37	2.18
													

### Seed Soluble Protein and Immunoreactivity

Whole seeds were ground to pass through a 20-mesh sieve. Soluble proteins were extracted in borate saline buffer (BSB) at flour: BSB = 1:10 (w/v). Flours were defatted and subjected to previously reported amandin cryoprecipitation methods (Su et al., 2015, 2017; Liu et al., 2017). Soluble protein was determined by Bradford and Lowry methods. Solubilized proteins were analyzed using electrophoresis and immunoassays employing mAbs 4C10 to assess conformational epitope immunoreactivity as described in Su et al. (2015).

### Aflatoxin

Whole seeds were ground to a fine powder as described above. A mixture of 5% almond kernel powder and 1.5% agar in 40 mL water was autoclaved and 10 mL sterile solution poured into 60-mm Petri dishes. Each Petri dish was inoculated with 200 spores of *Aspergillus flavus* and incubated at 30°C for 7 days as described by Gradziel et al. (2000). Samples were then derivatized and analyzed for aflatoxin by high-performance liquid chromatography with fluorescence detection as described by Goodrich-Tanrikulu et al. (1995) with four Petri dish samples being evaluated for each genotype.

### Oil Content and Composition

Total fat content and fatty-acid methyl esters (FAMEs) were determined according to the procedure of Garces and Mancha (1993). The FAMEs were identified based on retention times of known standards (Sigma, St. Louis). The presence of 17:0 as an internal standard allowed the calculation of the total lipids based on the area of the standard. Data were recorded on a dry-weight (DW) basis and analyzed using the SAS analysis of variance procedure for balanced data and the SAS REG procedure for regression analysis (SAS Institute, 1988) as previously described by Abdallah et al. (1998).

### Navel Orangeworm (NOW) Infestation

Fruits were collected from UCD research plots at Winters, CA and inspected visually to ensure no previous infestation by navel Orangeworm (NOW). A total of 24 nuts of each selection were tested as exposed kernels (shells broken to expose kernels). Samples were placed in individual plastic containers with 15 NOW eggs added and incubated at 25°C for 90 days. Proportion of samples containing mature NOW moths at the end after 90 days were recorded.

### Hull-Rot

Disease assessment was as described by Fresnedo-Ramírez et al. (2017). Fruit from each selection were harvested from UCD research plots at Winters, CA and stored at 4°C. Stored fruit were warmed to room temperature for 24 h prior to inoculation, surface sterilized for 30s by immersion in 10% bleach, rinsed in deionized water, and dried. A total of 24 unblemished hulls for each selection were placed in humidified plastic containers. Each fruit was inoculated with a 10 μL droplet containing conidia of *Monilinia fructicola.* (mixed field isolates) at a concentration of 2.5 × 104 spores per mL from 7 to 10-day-old cultures. Disease severity for each selection was calculated as the proportion of fruit with lesions greater than 3 mm. at 3 days after inoculation and incubation of the hulls in the humidified containers at room temperature.

## Results and Discussion

### Seed Soluble Protein and Immunoreactivity

Seed soluble protein and kernel mass are uniformly high for all traditional varieties and new releases while immunoreactivity was moderate to high for traditional varieties but ranged from less than one-half to almost double the “Nonpareil” standard in the more genetically diverse new releases (Table 1). Strong breeding selection for self-fertility and local adaptability (which would have included kernel mass) thus does not appear to reduce variability for immunoreactivity, allowing subsequent selection within commercially adapted germplasm for reduced immunoreactivity risk.

**FIGURE 1:**
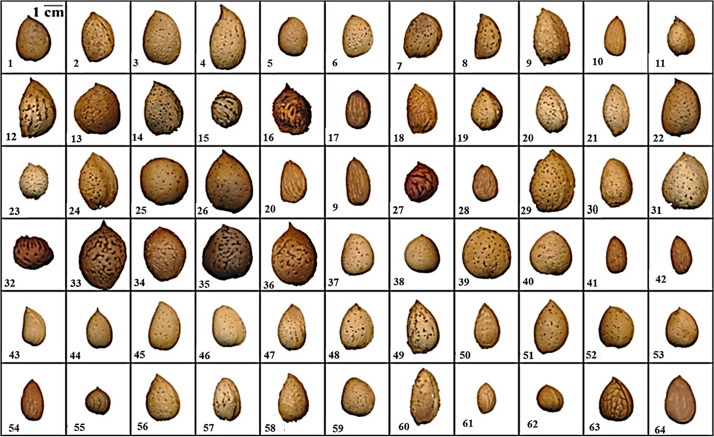
Nut and kernel morphologies for an intra- and interspecific almond breeding germplasm. (Identifying numbers refer to the first column of Table 1).

Variability for all traits evaluated, including size, shape, soluble protein content and ELISA immunoreactivity was documented in this diverse germplasm (Table 1 and Figure 1). Kernel mass, a critical commercial trait, ranges from 0.11 *g* to 2.08 *g*. All commercial varieties were approximately 1 *g* or greater, which has been shown to be an important threshold for optimizing orchard yield (Gradziel and Lampinen, 2013).

ELISA immunoreactivity values ranged from 0.26 to 2.18 times the level found in the “Nonpareil” standard, while soluble protein, an important trait in both processing and nutritional quality, ranges from 12.4 to 26.5 (*g*/100 *g*). The lower immunoreactivity scores were more strongly associated with interspecific hybridization lineages having peach or the wild almond species *P. argentea* or *P. webbii*. Velasco et al. (2016) have shown that while almond and peach are closely related and readily intercrossed, considerable trait differentiation has occurred between the species, suggesting fruit divergence long preceded domestication. The higher immunoreactivity scores were associated with hybridizations with *P. fenzliana*, which is generally considered to be one of the species from which cultivated almond was derived (Gradziel, 2011). No correlation was observed between almond seed size and either total soluble protein or amandin content. ELISA did show a general increase with increases in soluble protein content when only commercial varieties were analyzed. This positive association between amandin and immunoreactivity is consistent with previous reports analyzing a broader range of commercial varieties that identified amandin, also known as almond major protein (AMP), prunin, 11S globulin, and Pru du 6, as the major storage protein in commercial almond seed (Sathe et al., 2001). This relation does not hold up, however, within the species, interspecies hybrids and introgressed germplasm. Of the 15 genotypes showing ELISA values of approximately one-half or less of the “Nonpareil” standard, four are found in commercially desirable selections having an average kernel mass of approximately 1 *g* or greater. In addition to sizable reductions in immunoreactivity, examples of increased immunoreactivity are also evident, as in the commercial varieties “Chips” and “Sweetheart,” showing ELISA values of 1.68 and 1.73 of the “Nonpareil” standard, respectively.

All commercial varieties show ELISA values approaching or exceeding that of the “Nonpareil” standard with the exception of the Italian variety “Tuono.” “Tuono” is unique among Mediterranean and California varieties in that it is self-compatible and so self-fertile. Recent molecular analysis has demonstrated the source of this self-fertility was a natural introgression from *P. webbii* which is native in the regions of southern Italy were “Tuono” originated (Gradziel and Martínez-Gómez, 2013). Similarly, the soluble protein content of 17.14 for “Tuono” is unusually low for a commercial cultivar, being well below the 20 *g*/100 *g* level desired for some forms of processing.

Several advanced introgression breeding selections combine the desirable characteristics of sweet kernels with high mass and high soluble protein content with low immunoreactivity. These include the new releases #42, UCD,8–27 [Almond × *(P. webbii* × *P. persica)]BC3* (i.e., three consecutive backcrosses to almond), and selection #54, UCD,2–240 (“Nonpareil” × *P. webbii)BC3*. Both intraspecific breeding selections are currently being considered for release as improved varieties based on their desirable nut and kernel characteristics (Table 1 and Figure 1), self-fertility derived from peach and *P. webbii*, respectively, and high crop productivity.

### Aflatoxin

A 77% reduction in aflatoxin was observed in the introgression-derived “Sweetheart” variety when compared to the “Nonpareil” standard (Table 2). Even higher levels of aflatoxin were observed in the other traditional varieties. Field suppression of aflatoxin contamination in “Sweetheart” is further enhanced through improved resistance to pest and mold damage associated with aflatoxin production.

**TABLE 2:** Kernel oil quantity and quality along with susceptibility to aflatoxin contamination, hull-rot disease and Navel-orangeworm (NOW) infestation for the almond variety *Sweetheart* compared to six commercial variety standards.

	**Nonpareil**	**Sweetheart**	**Mission**	**Sonora**	**Chips**	**Kahl**	**Winters**
Total oil (% dry weight)	38.8 (0.3)	47.3 (1.2)	43.4 (1.2)	43.8 (2.3)	38.4 (1.7)	44.7 (1.8)	43.4 (0.6)
Oleic acid (%)	66.8 (0.8)	73.0 (1.3)	71.9 (2.3)	69.3 (2.3)	66.0 (4.3)	67.3 (1.1)	66.9 (0.4)
Aflatoxin (ug *g*-1 dry wt.)	0.17 (0.02)	0.04 (0.003)	0.20 (0.04)	0.25 (0.05)	0.24 (0.05)	0.31 (0.02)	0.22 (0.05)
Hull rot (%)	97.3 (8.8)	23.1 (6.9)	64.5 (6.7)	83.7 (6.1)	72.8 (5.5)	86.5 (5.3)	55.4 (7.2)
NOW (%)	79.5 (5.3)	4.1 (0.8)	39.8 (4.7)	64.1 (6.3)	48.4 (5.1)	56.8 (7.1)	81.3.8 (3.5)

“Sweetheart” is a UCD released commercial cultivar originating as a “Mission” almond by peach introgression line (“Mission” × *P. persica*)BC3 in an effort to transfer self-fertility from peach (Gradziel et al., 2001). While not expressing sufficiently high levels of self-fruitfulness to be commercially distinct, “Sweetheart” possesses an exceptionally high oil content as well as quality as demonstrated by its very high oleic acid content (Table 2) placing it in a premium roasting-quality category with the Spanish variety “Marcona” (Gradziel et al., 2013). “Sweetheart” is also exceptional in that, since its release in 2007, very few positive findings for aflatoxin contamination have been reported in commercial shipments. Early analysis by Gradziel et al. (2000) had shown significantly lower levels of aflatoxin production following inoculation under controlled laboratory conditions. More recent studies have shown that this variety also shows higher resistance to hull-rot as well as NOW infestation (Table 2).

**FIGURE 2:**
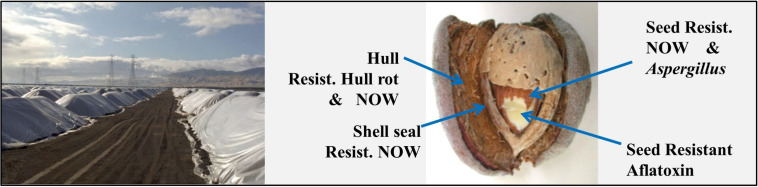
In-field, bulk storage of almond fruit (hulls, shells, and kernels) under plastic tarps (left). Structure of the mature, dried almond fruit identifying the major components: hull, shell, and seed/seedcoat, along with associated resistances as identified in the “Sweetheart” variety (right).

Improved performance in unrelated traits is not unusual in interspecific introgressions because of the inherently higher genetic and so trait variability compared to the highly inbred (Gradziel, 2017) and so trait limited nature of traditional Californian varieties (Gradziel et al., 2001). In “Sweetheart,” however, these traits appear to be complementary in reducing the overall risk of aflatoxin contamination. Under field conditions, *Aspergillus flavus* infection usually occurs following kernel damage by NOW, where infestation acts to inoculate the normally shell-protected kernel and where subsequent feeding creates a suitable environment for *A. flavus* growth and aflatoxin development (Hamby et al., 2011). Kernel infestation/infection can occur in the field from the time of fruit maturity (where the flesh or hull splits exposing the almond nut), to field harvest, and again during storage prior to hulling and shelling. In almond, as with other *Prunus* or “stone-fruit” species, the mesocarp develops into the hull or fruit flesh and the endocarp develops into the shell enclosing the nut/kernel, which is the seed with or without tegument/seed coat. Because of the size of the 1 billion kg. (kernel meat) crop, fruit are air-dried in the field and held in bulk storage for several months or more (Figure 2). When properly dried, nuts are relatively resistant to new NOW infestation because the 1st instar larvae are very delicate and particularly vulnerable to desiccation or starvation before it can access the nut kernel (Hamby et al., 2011). The occurrence of hull-rot during storage, however, acts to both macerate and hydrate hull tissue, making it much more vulnerable to NOW infestation. Under field conditions, the multiple barriers found in the “Sweetheart” almond, including increased resistance to NOW as well as hull-rot development, the reduced tendency for aflatoxin production and a highly sealed shell (Figure 2) have resulted in a high level of field resistance to this economically important insect-disease complex.

### Soil-Born Contaminants

Soil-borne contaminants are an inevitable consequence of commercial off-ground harvest practices. Improved harvest methods, such as catch-frame harvesting, avoid the risk of soil-contamination but require novel fruit and nut traits in order to be commercially feasible. Required traits are available within the enriched, interspecies-introgressed breeding germplasm that are compatible with current and future harvest needs.

**FIGURE 3:**
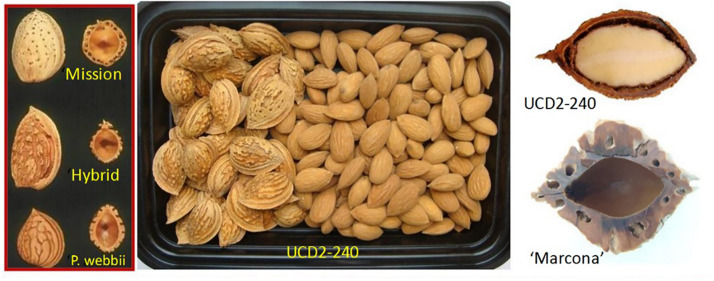
Longitudinal and cross-section images of shells of the wild almond species *P. webbii* (#63 in Table 1 and Figure 1), the thin “paper”-shelled California “Mission” variety (#19), their interspecies hybrid, and the interspecies introgression selection UCD2-240 (#54) along with a shell cross-section of the Spanish variety “Marcona” for comparison (#25 in Table 1 and Figure 1).

A problem with soil contaminants such as *Salmonella, E. coli* and pesticide residues is the difficulty in defining safe concentrations and so even detection of trace levels can lead to crop rejection. Avoiding contamination remains the most effective strategy for ensuring food safety. Like peach, the almond kernel is enclosed in a lignified endocarp or shell (Figure 3), which, if highly sealed, confers protection from insect infestation and mold infection. Unfortunately, an important post-harvest role of the shell is to facilitate the uptake of moisture for seed hydration/germination. Danyluk et al. (2008) have demonstrated that this moisture uptake pathway also provides a ready conduit for the entrance of bacteria and contaminated water.

**FIGURE 4:**
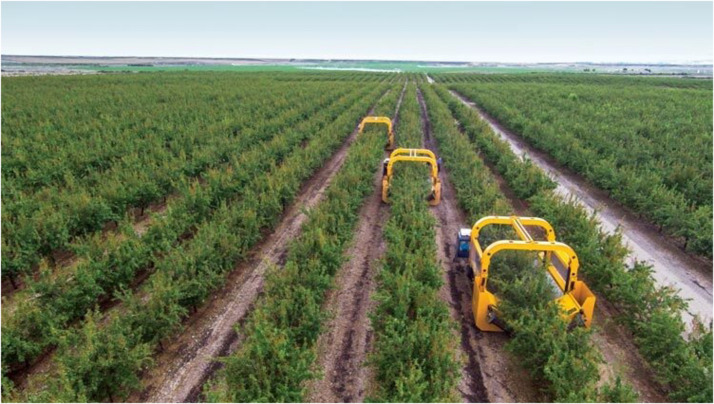
Catch-frame harvesting of almonds in Spain using specialized equipment integrating tree-shakers and collection frames for catching harvested fruit (hulls plus nuts) before they hit the ground.

A solution currently being pursued by the California almond industry is the use of catch-frame harvesting as currently practiced for pistachio in California and some orchards in Spain (Figure 4) because it avoids off-ground nut harvest with its high risk of soil contamination. In current practice, California almonds are shake-harvested to the orchard floor and allowed to dry in the Central Valley’s warm, dry environment to kernel moisture levels of 7% or less to suppressed post-harvest disease. Dried fruit (hulls plus nuts) are then collected and bulk-stored until hull removal (hulling) and shelling in specialized industrial facilities. While off-site drying is feasible with the relatively limited production of California pistachio and Spanish almond, it presents huge technical challenges for the 4 billion kg. almond crop (2 billion kg. in hulls, 1 billion kg. in shells, and 1 billion kg. in kernel-meats). In-field hulling at harvest would reduce post-harvest handling by half and allow the vegetative hulls to be reincorporated into orchard soils in a more sustainable manner. Unlike Spanish almonds where the thick, highly lignified shells typically constitute about two thirds of the nut mass (Figure 3), California almonds have thin, “paper” shells with improved harvest index and shelling efficiency. The fragile nature of paper-shells results in unacceptable levels of nut and kernel damage with mechanically intensive in-field hulling, while the highly lignified Spanish-type shells dramatically reduce harvest efficiency and would require extensive retooling of industrial shelling equipment. Wild almond species such as *P. argentea, P. bucharica*, and *P. webbii* (#55, 61, and 63 in Figure 1) possess a thin, highly lignified shell that confers high structural strength while allowing a high kernel-to-nut “crack-out” ratio. This trait has proven highly heritable in certain *P. webbii* introgression lines allowing the development of California-adapted almonds possessing thin yet highly lignified *P. webbii*-type shells. An example can be seen in the previously discussed low-aflatoxin selection UCD,2-240 (#54 in Figures 1, 3 and Table 1). Combining good kernel size and quality with a durable, highly-sealed shell having a kernel-to-nut crack-out ratio of 70%, UCD,2-240 is currently undergoing grower field testing as a candidate for almond catch-frame harvest.

## Conclusion

Selection during crop domestication for desirable traits such as large, non-bitter seed and uniform harvest to facilitate cultivation also results in a loss of genetic diversity and so trait variability. Results show that the absence of traits required for evolving market and agronomic needs are often the consequence of diminished genetic diversity and are not pleiotropic effects of the selection for specific commercial traits. For crops such as almond, possessing an extensive and diverse germplasm within its wild relatives and a relatively flexible characterization of crop-ideotype, a re-domestication process can develop commercial varieties with an enriched germplasm and so expanded opportunities for novel trait selection. These findings suggest that many of the relatively recently identified food safety threats may not be an inherent hazard of the crop but rather are a consequence of the limits imposed by the initial domestication events and later production practices. Where appropriate germplasm remains accessible, modern breeding programs can sometimes redo the domestication process, which, if properly focused, can provide a more effective selection for traits allowing agronomically viable solutions to modern food safety challenges.

## Data Availability Statement

The datasets generated for this study are available on request to the corresponding author.

## Author Contributions

The author confirms being the sole contributor of this work and has approved it for publication.

## Conflict of Interest

The author declares that the research was conducted in the absence of any commercial or financial relationships that could be construed as a potential conflict of interest.

## Dedication

This manuscript is dedicated to Shridhar K. Sathe (October 30, 1950–April 4, 2019), the Robert O. Lawton Distinguished Professor and Hazel K. Stiebeling Professor in the College of Human Sciences, Florida State University, in recognition of his pioneering work on tree nut allergy identification and control.
